# Identification of the NTL Gene Family in *Beta vulgaris* L. and Functional Role of *BvNTL2* in Drought Resistance

**DOI:** 10.3390/plants14101528

**Published:** 2025-05-20

**Authors:** Ziqi Fan, Yanni Xu, Yaqing Sun, Ningning Li, Shaoying Zhang, Guolong Li

**Affiliations:** College of Agriculture, Inner Mongolia Agricultural University, Hohhot 010018, China; polarisfzq@outlook.com (Z.F.); octansxyn@163.com (Y.X.); syaqing@imau.edu.cn (Y.S.); lnn@imau.edu.cn (N.L.); syzh36@aliyun.com (S.Z.)

**Keywords:** *Beta vulgaris* L., membrane-bound transcription factors, NTL gene family, transgenic *Arabidopsis*, drought resistance analysis

## Abstract

NAC transcription factors form a plant-specific family essential for growth, development, and stress responses. NTLs, a subfamily of the NAC transcription factor family, belong to the membrane-bound transcription factors (MTFs). These proteins contain transmembrane domains that enable rapid nuclear translocation in response to environmental stimuli, thereby regulating target gene expression. As a major sugar crop, sugar beet is primarily cultivated in arid and semi-arid regions, where drought stress significantly impairs yield and quality, underscoring the urgent need to improve its drought tolerance. This study identified the NTL gene family in sugar beet and analyzed its gene structure, evolutionary relationships, cis-regulatory elements, drought-induced expression patterns, and *BvNTL2*’s role in drought resistance. The *BvNTLs* family comprises five members located on five distinct chromosomes. Their promoters harbor cis-regulatory elements related to ABA and drought stress, and their expression is drought-responsive. Under drought stress, *BvNTL2* translocates to the nucleus, where its transmembrane domain is cleaved, resulting in its direct nuclear localization. Functional validation in *Arabidopsis* demonstrated that *BvNTL2* overexpression enhances drought tolerance by increasing antioxidant enzyme activities and promoting the expression of ABA-related genes. This study highlights *BvNTL2* as a promising candidate gene for the genetic improvement of drought-resistant sugar beet.

## 1. Introduction

The NAC transcription factors form one of the largest plant-specific transcription factor families [1], named after three subfamilies: NAM, ATAF, and CUC [2,3]. NAC proteins have diverse and essential functions in plants, regulating development, participating in signal transduction, and responding to abiotic stresses [4]. NAC proteins comprise a highly conserved N-terminal NAM domain and a highly variable C-terminal region. The highly conserved N-terminal NAM domain, typically 150 amino acids long, is subdivided into five subdomains (A–E) [5]. This domain facilitates nuclear localization, DNA binding, and homo- or heterodimer formation with other NAC proteins [4]. In contrast, the highly variable C-terminal region plays a crucial role in transcriptional regulation and determines NAC protein multifunctionality [6,7]. Membrane-bound transcription factors (MTFs) have emerged as a research focus in recent years [8]. Under normal conditions, MTFs are synthesized as full-length proteins in the cytoplasm, remaining anchored to the membrane in a dormant state. In response to environmental stimuli, the full-length protein is cleaved from the membrane and translocates to the nucleus to perform its function. In contrast to typical free transcription factors, MTFs require activation by external signals, such as hormones or stresses. This activation mechanism ensures that target gene expression is triggered only under specific conditions, enhancing response precision [9]. Additionally, since MTFs are primarily membrane-localized, their proximity to signal receptors enables faster detection and response to external stimuli [10]. This characteristic allows MTFs to rapidly regulate target gene transcription, providing essential support for plants to adapt to changing environments [11]. NTM1 (NAC with Transmembrane Motif 1), also known as NTL (NTM1-like), is a typical member of the NAC transcription factor family, characterized by a transmembrane domain (TM) near its C-terminus. NTL genes have been identified in various species, including eighteen in *Arabidopsis thaliana*, five in *Oryza sativa* [12], six in *Zea mays* [13], fifteen in *Glycine max* [14], and twelve in *Solanum lycopersicum* [15]. Studies have demonstrated that NTL genes play key roles in plant growth, development, abiotic stress regulation, and defense responses [16]. For instance, *AtNTM1* is the first NAC transcription factor identified with a transmembrane structure, localized on membranes surrounding the nucleus, including the nuclear membrane and endoplasmic reticulum. It inhibits cell division by inducing cyclin-dependent kinase (CDK) inhibitor gene expression (KRPs) and suppressing histone H4 gene transcription [17]. *AtNTL8* regulates seed germination by modulating GA-mediated salt signaling [18]. Similarly, *AtNTM2* employs a similar adaptation strategy, integrating auxin signaling and salt stress during seed germination to ensure germination occurs only under favorable conditions [19]. Under drought and dark stress, the *AtNTL3* gene undergoes TM cleavage, translocating to the nucleus, where it promotes drought stress response through a feed-forward loop involving NAP and AREB1. *AtNTL6* localizes to the plasma membrane and is induced by ABA, low temperature, salt, and drought. Overexpression of a truncated form of *AtNTL6* enhances drought tolerance in transgenic plants. *TaNTL1* localizes to the endoplasmic reticulum and positively regulates drought tolerance in plants [20]. *LiNAC014* enhances thermotolerance by sensing high temperatures, translocating to the nucleus, and activating the DREB2-HSFA3 module [21]. *AtNTL11* positively regulates flavonoid biosynthesis-related genes and enhances plant response to strong light stress by promoting anthocyanin accumulation [22]. *ANAC017* significantly induces ABA and oxidative stress response genes, enhancing plant tolerance to waterlogging through retrograde signaling from mitochondria [23,24]. *AtNTL9* significantly induces SA biosynthesis enzyme genes and, by participating in SA synthesis and signaling, leads to stomatal closure, preventing pathogen entry and enhancing disease resistance [25]. The late blight pathogen *Phytophthora infestans* RxLR effector PITG_03192 (Pi03192) interacts with them on the endoplasmic reticulum membrane, preventing their translocation to the nucleus, thereby increasing susceptibility to the pathogen [26]. These results suggest that the NTL family plays a crucial role in enhancing plant stress resistance, offering new insights for improving drought tolerance in sugar beet.

*Beta vulgaris* L. is a key sugar crop in northern China, primarily cultivated in the North China and Northwest regions. These regions predominantly belong to arid and semi-arid climate zones, characterized by low annual precipitation and uneven seasonal distribution. In recent years, to minimize competition with staple crops and optimize crop rotation, some sugar beet cultivation areas have been relocated to drier regions. Moreover, water restriction policies for ecological conservation further intensify drought stress, posing a major challenge to the sustainable development of the sugar beet industry in key production regions. The cultivated area of sugar beet declined steadily from 244,800 hectares in 2002 to just 170,000 hectares by 2022. In response to drought stress, plants regulate gene expression through complex molecular mechanisms to enhance stress tolerance. Transcription factors, particularly membrane-bound transcription factors (MTFs), play a pivotal role in this process by perceiving environmental signals and rapidly triggering responses. Although the NTL family has been relatively well studied in other plant species, research on its functions in sugar beet and related species such as spinach and amaranth remains limited, particularly regarding its role in drought stress responses. Thus, a comprehensive study of the NTL gene family in sugar beet is essential for elucidating drought resistance mechanisms and facilitating drought-resistant breeding.

Based on the 52 NAC transcription factor genes identified in our laboratory [27], we characterized the NTL gene family in sugar beet and analyzed its physicochemical properties, chromosomal localization, gene structure, evolutionary relationships, and expression patterns. Furthermore, we examined the role of *BvNTL2* in drought stress response. This study provides a theoretical basis for further functional research on the sugar beet NTL gene family, offering insights into drought resistance mechanisms and supporting the development of drought-resistant candidate genes.

## 2. Results

### 2.1. Identification and Prediction of the Physicochemical Properties of BvNTLs Family Members

**Figure 1:**
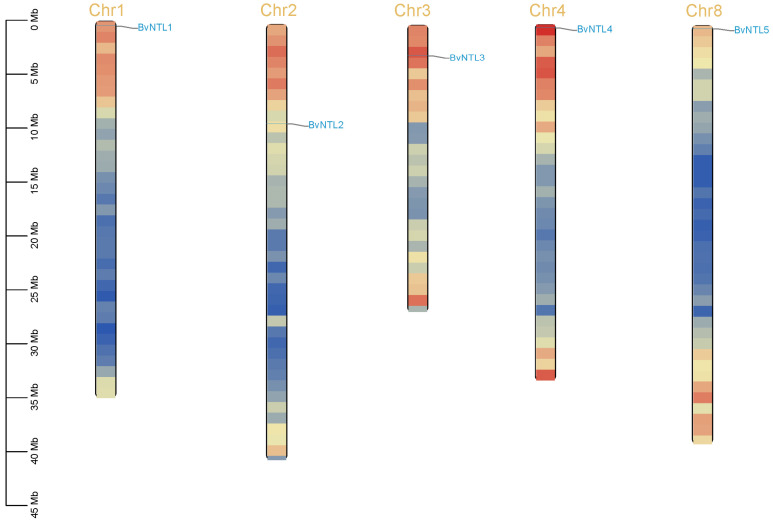
Chromosomal locations of *BvNTLs*.

Five *BvNTLs* genes were identified in the sugar beet genome. Chromosomal localization analysis showed that they are unevenly distributed across five chromosomes. They were named *BvNTL1* to *BvNTL5* according to their chromosomal positions (Figure 1). Protein analysis revealed that BvNTLs proteins range from 383 to 608 amino acids in length, with BvNTL1 being the shortest and BvNTL3 the longest. The isoelectric points of BvNTLs proteins range from 4.57 (BvNTL4) to 8.70 (BvNTL1). Hydrophilicity analysis confirmed that all BvNTLs proteins are hydrophilic. Subcellular localization predictions indicated that all BvNTLs proteins, except *BvNTL4* (localized to the endoplasmic reticulum), are nuclear-localized, consistent with typical transcription factor characteristics.

### 2.2. BvNTLs Family Gene and Protein Structure Analysis

**Figure 2:**
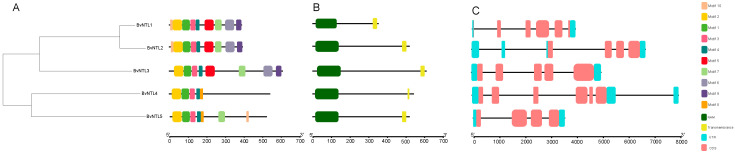
Gene structure and conserved domain of *BvNTLs* in sugar beet. (**A**) Evolutionary relationship and conserved motif. (**B**) Conserved domain. (**C**) Gene structure.

Analysis of conserved motifs, domains, and gene structures in the *BvNTLs* gene family revealed that all members contain over five conserved motifs, which show high conservation among family members (Figure 2A). Additionally, conserved domain predictions indicate that *BvNTLs* genes harbor both an NAM domain and a transmembrane domain (Figure 2B), with the latter located at the C-terminal region. This suggests that the *BvNTLs* gene family is part of the membrane-bound transcription factors (MTFs). Gene structure analysis (Figure 2C) revealed variations in the number of exons and introns among *BvNTLs* genes, with exon numbers ranging from four to six and intron numbers from three to six Specifically, *BvNTL1* and *BvNTL2* each contain five introns, with *BvNTL1* having three exons and *BvNTL3* having five. *BvNTL5* has the highest number of exons (six) and introns (six). This structural variability may be linked to the functional diversification of *BvNTLs* genes during evolution.

### 2.3. Prediction of Cis-Acting Elements in the Promoter Region of the BvNTLs Family

**Figure 3:**
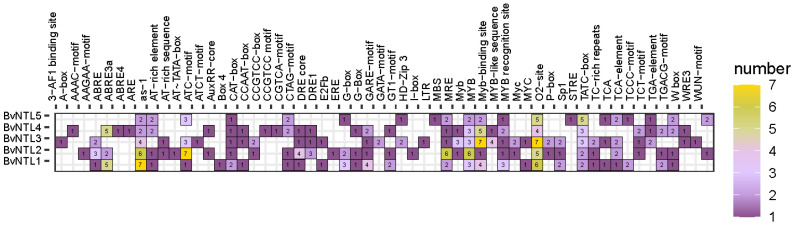
Cis-acting elements of *BvNTLs* genes displayed graphically.

To further investigate the function of *BvNTLs* genes in sugar beet, cis-acting elements in the 2000 bp upstream promoter region were predicted and analyzed (Figure 3). The results showed that 42 cis-acting elements were identified in the promoter regions of the *BvNTLs* gene family, categorized into three main groups: plant hormone response, abiotic stress response, and development-related signaling (Figure 3). Plant hormone response elements, such as ABRE (abscisic acid), TGA (auxin), GARE-motif (gibberellin), and CGTCA-motif (jasmonic acid methyl ester), were widely distributed in *BvNTLs* genes, with ABRE and ABRE3a occurring most frequently. Abiotic stress response elements included DRE (drought response), LTR (low temperature response), ARE (antioxidant response), and MBS (drought-induced response). Development-related signaling elements included mainly light-responsive elements, such as Box4, G-box, and GT1-motif, as well as the CAT-box element associated with meristem expression.

### 2.4. Collinearity Analysis of NTLs in Multiple Species

**Figure 4:**
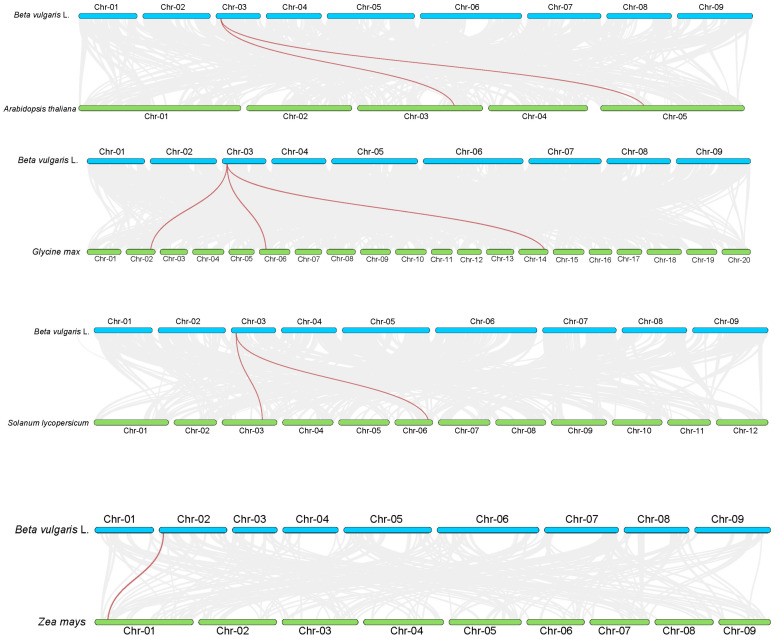
Collinearity analysis of *BvNTLs* between *Beta vulgaris* L. and four other species.

In a cross-species synteny analysis with *Arabidopsis thaliana*, *Solanum lycopersicum*, *Glycine max*, and *Zea mays* (Figure 4), *BvNTL3* showed synteny with *Arabidopsis*, tomato, and soybean, while *BvNTL2* exhibited synteny only with maize. This suggests that these two genes may have retained more conserved functions during evolution. However, synteny analysis in sugar beet revealed no syntenic relationship among the *BvNTLs* genes, suggesting that functional diversification and genomic variation of the *BvNTLs* gene family occurred during specific evolutionary processes.

### 2.5. Phylogenetic Tree of the BvNTLs Family

**Figure 5:**
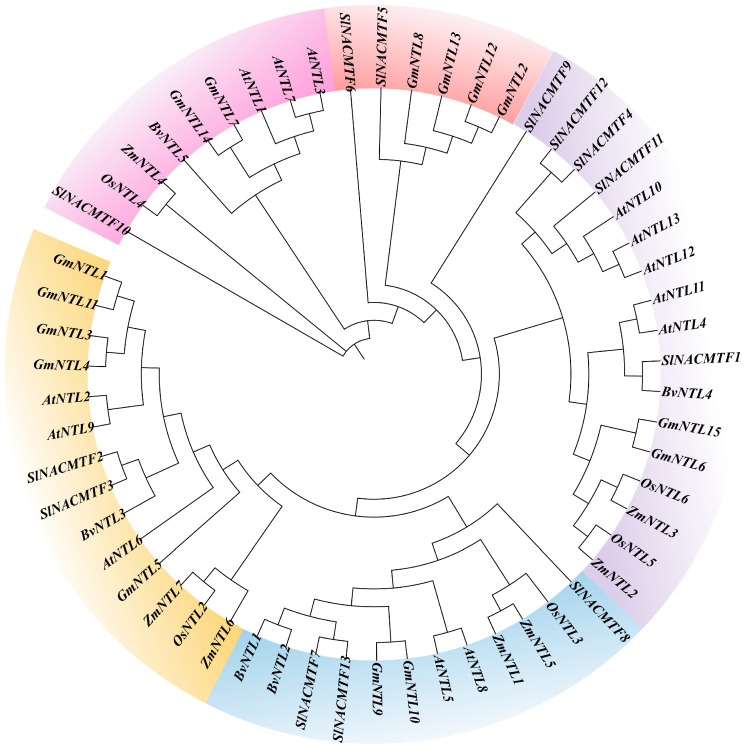
Phylogenetic tree of the *BvNTLs* family. At—Arabidopsis thaliana; Bv—Beta vulgaris L.; Gm—Glycine max; Os—Oryza sativa; Si—Solanum lycopersicum; Zm—Zea mays.

Phylogenetic analysis of the NTL gene family in *Beta vulgaris* L., *Arabidopsis thaliana*, *Oryza sativa*, *Glycine max*, *Zea mays*, and *Solanum lycopersicum* (Figure 5) revealed that the NTL genes did not cluster strictly by species classification. Instead, they formed distinct evolutionary branches based on gene sequences and functional relatedness. The close relationship between the NTL genes of *Beta vulgaris* L., *Arabidopsis thaliana*, and *Solanum lycopersicum* suggests that these genes may share similar functions or evolutionary origins.

### 2.6. Expression Analysis of BvNTLs Members in Sugar Beet Leaves

**Figure 6:**
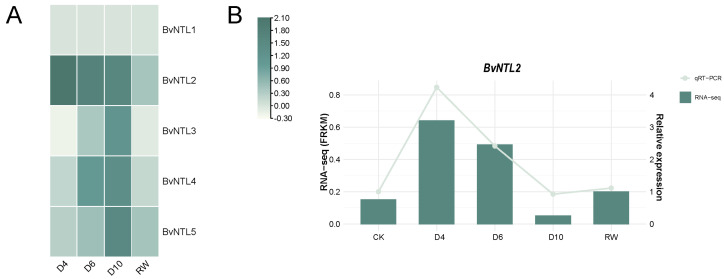
Expression patterns of *BvNTLs.* (**A**) Expression patterns of *BvNTLs* under drought stress based on transcriptomic data. (**B**) qRT-PCR validation of *BvNTL2* expression under drought stress. Drought stress was simulated by reducing soil water content to 40% of field capacity.

Transcriptome data of the *BvNTLs* gene family under various drought treatments were analyzed using TBtools (version 2.1.52). The results showed that the expression levels of most *BvNTLs* genes were significantly upregulated on the 10th day of drought stress treatment (Figure 6A). However, *BvNTL2* exhibited a significant increase in expression as early as the fourth day of drought stress. To validate the transcriptome data, the expression pattern of *BvNTL2* was further examined using quantitative reverse transcription PCR (qRT-PCR) (Figure 6B). The validation results confirmed that the expression pattern of *BvNTL2* was consistent with the RNA-Seq data. These results suggest that *BvNTL2* is highly sensitive to drought stress and responds rapidly early in drought conditions. Its specific role in drought stress response requires further investigation.

### 2.7. Functional Analysis of BvNTL2

#### 2.7.1. Subcellular Localization Analysis of BvNTL2

**Figure 7:**
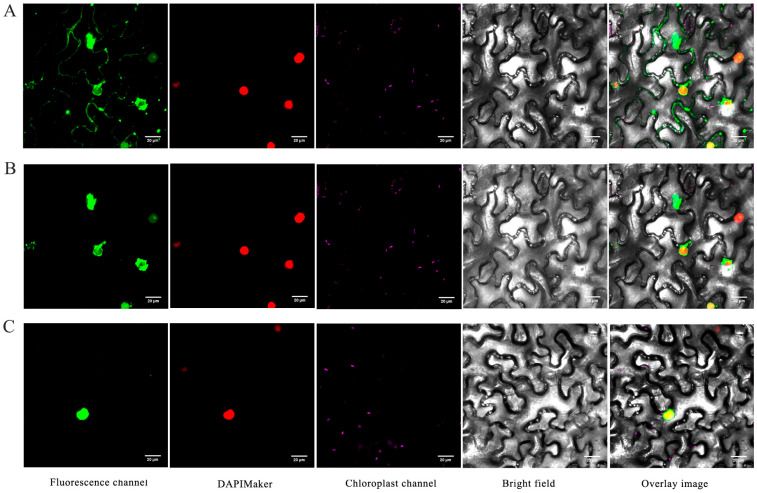
Subcellular localization of *BvNTL2* in the epidermis of tobacco leaves. Bar = 20 µm. (**A**) Subcellular localization of *BvNTL2* under normal water conditions. (**B**) Subcellular localization of *BvNTL2* under drought stress conditions. (**C**) Subcellular localization of *BvNTL2* lacking the transmembrane domain.

In order to investigate the subcellular localization and drought-responsive behavior of *BvNTL2*, transient transformation technology was employed in tobacco leaves. Fluorescence was observed using a laser confocal microscope with excitation at 488 nm and emission at 510 nm. Under normal conditions, fluorescence was detected in both the plasma membrane and cytoplasm (Figure 7A). Under drought stress, nuclear fluorescence of *BvNTL2* was significantly enhanced (Figure 7B). Moreover, a truncated form of *BvNTL2* lacking the transmembrane domain was localized exclusively to the nucleus (Figure 7C). These findings suggest that *BvNTL2* acts as a membrane-bound transcription factor that translocates to the nucleus upon drought stress to regulate downstream gene expression.

#### 2.7.2. Drought Resistance Identification of *BvNTL2* Overexpression

**Figure 8:**
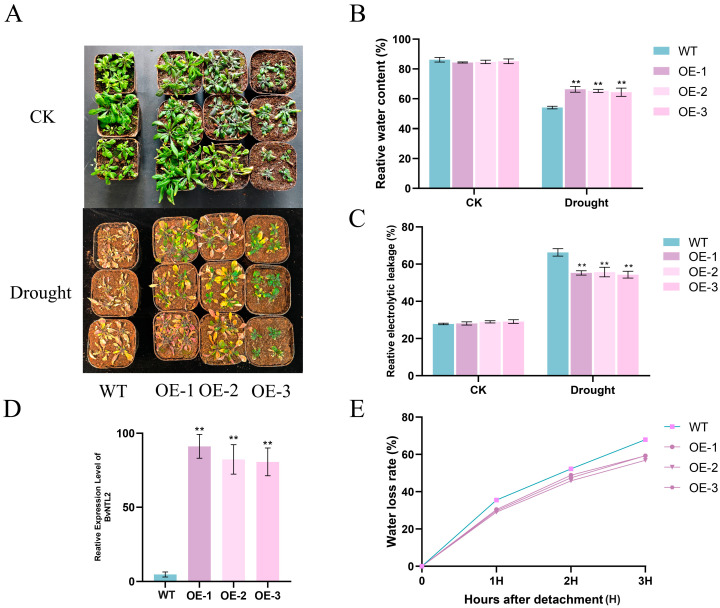
*BvNTL2* increased drought tolerance by plants. (**A**) Phenotypes of transgenic *Arabidopsis* and wild-type (WT) plants before and after drought treatment. (**B**) Relative water content of transgenic *Arabidopsis* overexpressing plants and WT plants before and after drought treatment. (**C**) Relative electrolyte leakage by transgenic *Arabidopsis* overexpressing plants and WT plants before and after drought treatment. (**D**) Determination of relative expression of transgenic lines. (**E**) Water loss rates by transgenic *Arabidopsis* overexpressing plants and WT plants. The data represent the mean ±standard deviation (SD) (n = 3, each replicate containing 3 plants). ** *p* ≤ 0.01.

To explore the role of *BvNTL2* in drought stress, we generated *Arabidopsis* lines overexpressing *BvNTL2* via genetic transformation (Figure 8). Under normal conditions, *BvNTL2*-overexpressing lines showed no significant differences from wild-type (WT, *Arabidopsis thaliana* Col-0) plants. After two weeks of drought treatment, most WT plants developed wilted leaves, whereas *BvNTL2*-overexpressing plants maintained more green foliage. Additionally, the OE-3 line exhibited consistently smaller growth, possibly due to variations in the T-DNA insertion site, which may affect the expression of nearby genes and lead to growth differences among individuals. The relative water content was significantly higher in *BvNTL2*-overexpressing lines than in WT plants. WT plants exhibited significantly higher relative electrolyte leakage than *BvNTL2*-overexpressing lines. The water loss rate was also higher in WT plants than in *BvNTL2*-overexpressing lines.

#### 2.7.3. Impact of *BvNTL2* Overexpression on Physiological Traits Associated with Stress Tolerance

**Figure 9:**
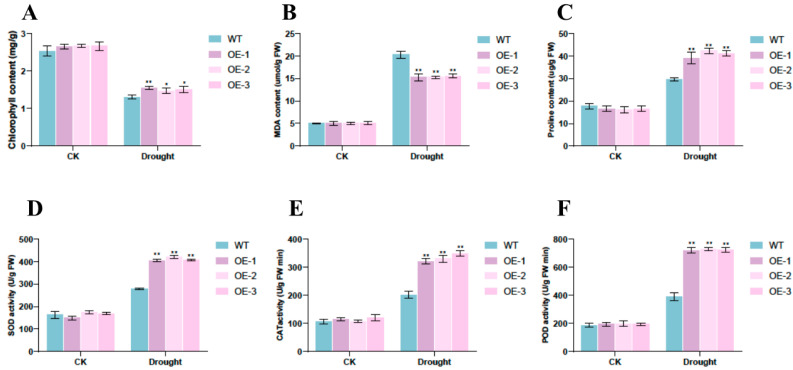
Physiological changes in transgenic *Arabidopsis* plants and WT plants under normal conditions and drought stress. (**A**) Chlorophyll content; (**B**) malondialdehyde (MDA) content; (**C**) proline content; (**D**) superoxidase dismutase (SOD) activity; (**E**) catalase (CAT) activity; (**F**) guaiacol peroxidase (POD). The data represent the mean ± standard deviation (SD) (n = 3, each replicate containing 3 plants). ** *p* ≤ 0.01, * *p* ≤ 0.05.

Prior to drought treatment, chlorophyll content, MDA levels, and proline accumulation were comparable between WT and *BvNTL2*-overexpressing lines, with no significant differences in antioxidant enzyme activity. Chlorophyll content decreased in all lines after drought stress, but the reduction was smaller in *BvNTL2*-overexpressing lines (51%) than in the wild type (56%). Similarly, malondialdehyde (MDA) levels were 27% lower in the overexpressing lines compared to the wild type. In addition, proline levels and the activities of SOD, POD, and CAT were significantly higher in the overexpressing lines (Figure 9). These findings suggest that *BvNTL2* enhances drought tolerance by increasing antioxidant enzyme activities.

#### 2.7.4. Impact of *BvNTL2* Overexpression on the Expression of Stress-Responsive Genes

##### Impact of *BvNTL2* Overexpression on the Expression of ABA Biosynthesis-Related Genes

**Figure 10:**
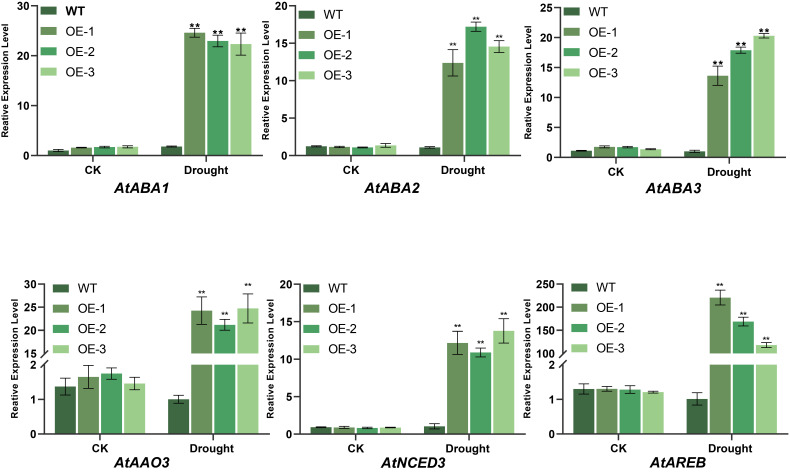
Expression of ABA biosynthesis-related genes in transgenic *Arabidopsis*. The data represent the mean ± standard deviation (SD) (n = 3, each replicate containing 3 plants). ** *p* ≤ 0.01.

Under drought stress, the expression of ABA biosynthesis-related genes *AtABA1*, *AtABA2*, *AtABA3*, *AtAAO3*, and *AtNCED3* was significantly increased in *BvNTL2*-overexpressing *Arabidopsis* lines, reaching levels approximately 20 times higher than in the WT. Moreover, the transcription factor *AtAREB* was strongly upregulated, with expression increasing by up to 200-fold (Figure 10). These results suggest that *BvNTL2* may improve drought tolerance by activating the ABA signaling pathway.

Impact of *BvNTL2* Overexpression on the Expression of Drought Stress-Related Genes

**Figure 11:**
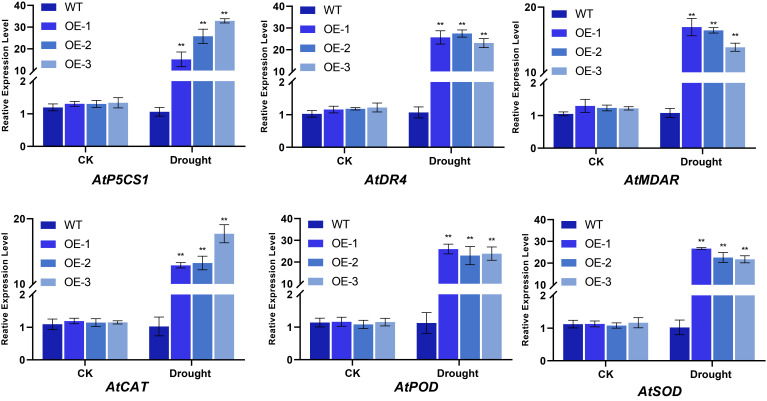
Expression of drought stress-related genes in transgenic *Arabidopsis*. The data represent the mean ± standard deviation (SD) (n = 3, each replicate containing 3 plants). ** *p* ≤ 0.01.

Under drought conditions, the expression of drought-responsive genes (*AtP5CS1*, *AtRD4*, and *AtMDAR*) and antioxidant enzyme-related genes (*AtCAT*, *AtPOD*, and *AtSOD*) was significantly higher in *BvNTL2*-overexpressing *Arabidopsis* lines than in the WT, with increases ranging from 10- to 30-fold. (Figure 11). The marked upregulation of *AtP5CS1* and *AtDR4* suggests that *BvNTL2*-overexpressing lines adapt to drought stress by promoting proline biosynthesis and modulating stress-responsive gene expression. Furthermore, the marked upregulation of antioxidant enzyme genes suggests that *BvNTL2* overexpression enhances antioxidant capacity, facilitating the scavenging of reactive oxygen species (ROS) produced under drought stress.

## 3. Discussion

### 3.1. The Relationship Between the NTL Gene Family and Drought Resistance in Sugar Beet

Transcription factors are abundant in plants, with approximately 10% localized to cellular membranes [28]. NTLs are a plant-specific subfamily of NAC transcription factors distinguished by a unique transmembrane domain that undergoes proteolytic cleavage in response to environmental and developmental signals, thereby facilitating rapid regulation of target gene expression. Compared with other NAC transcription factors, NTLs play an essential role in plants owing to their membrane-associated specificity. NTLs are highly conserved across higher plants [16], suggesting their role as key signal transducers in plant development and stress response pathways. However, research on NTLs in sugar beet is limited and their functional roles and regulatory mechanisms remain largely unexplored.

Five members of the *BvNTL* gene family were identified in sugar beet and designated *BvNTL1* through *BvNTL5*. These genes are located on five distinct chromosomes in the sugar beet genome, displaying an asymmetric distribution pattern. This asymmetric distribution may result from differential gene duplication and mutation events during the evolutionary history of the sugar beet genome [29]. The chromosomal dispersion of these genes may contribute to functional diversification, promoting subfunctionalization or neofunctionalization of family members, and enabling them to carry out distinct biological functions in plant development and stress adaptation [30]. Among family members, variation in amino acid length may suggest functional divergence. Longer proteins often contain additional domains that enable interactions with a wider range of transcription factors or target genes, thus conferring more complex regulatory potential. Differences in isoelectric points and hydrophilicity reflect variation in charge properties, which may influence subcellular localization and functional roles. Proteins with higher isoelectric points are often associated with processes involving the plasma membrane or nuclear envelope [31].

Variation in exon and intron numbers may be closely linked to the evolution of functional diversity [32], allowing genes to adopt distinct roles in plant growth, development, and stress responses. Multiple cis-acting elements were identified in the promoter regions of these genes, acting as key regulatory nodes within gene networks that control gene expression. Promoter analysis revealed 42 distinct types of cis-acting elements, which primarily fall into three categories: hormone response, abiotic stress response, and development-related signals. Among them, hormone-responsive elements—especially ABRE (ABA-responsive element) and ABRE3a—were widely and abundantly distributed across the promoters. As abscisic acid (ABA) is a key regulator of abiotic stress responses in plants [33], these findings suggest that the *BvNTLs* gene family may play a critical role in ABA signaling, especially under drought and other stress conditions. Cross-species synteny analysis showed that only *BvNTL2* and *BvNTL3* exhibited syntenic relationships, indicating genomic conservation and suggesting that these two genes may share conserved evolutionary functions, particularly in drought response [34]. In contrast, no syntenic relationships were found among *BvNTLs* genes within the sugar beet genome, possibly due to genome rearrangements, gene duplications, or species-specific adaptations during sugar beet evolution [35]. Transcriptome data showed that most *BvNTLs* genes were significantly upregulated after 10 days of drought treatment, with *BvNTL2* displaying sustained high expression from the early stages of drought stress. These transcriptomic results were validated by qRT-PCR, confirming the expression patterns and highlighting the role of these genes in sugar beet’s drought stress response. Furthermore, the results support the hypothesis that *BvNTL2* enhances drought tolerance in beet by modulating ABA signaling and other stress-responsive pathways.

### 3.2. Subcellular Localization and Functional Analysis of BvNTL2 in Drought Tolerance

Membrane-bound transcription factors typically contain one or more transmembrane domains at their C-terminus, which affect both their transcriptional activity and subcellular localization. The absence of these transmembrane domains promotes nuclear localization, which is critical for rapid stress response. Studies have shown that deletion of the transmembrane domain in TaNTL1 results in nuclear localization. Under PEG6000 or ABA treatment, part of the protein translocates from the plasma membrane to the nucleus [20]. OsNTL2 is expressed in both the plasma membrane and nucleus; however, after transmembrane domain deletion, the protein is detected exclusively in the nucleus [36]. BnaNTL1 is localized to the endoplasmic reticulum (ER) membrane, but deletion of its transmembrane domain or ER stress triggers relocation of the signal from the ER membrane to the nucleus [37]. NTL6 is primarily localized to the plasma membrane, but deletion of its transmembrane domain confines the signal to the nucleus. Moreover, ABA and cold treatments rapidly induce translocation of NTL6 from the plasma membrane to the nucleus [9]. In this study, BvNTL2 was predominantly localized to the nucleus, with minor presence at the plasma membrane. This localization may be due to a nuclear localization signal (NLS) near its C-terminal region, around amino acid position 330 [38]. Under simulated drought stress, nuclear fluorescence signals increased. When BvNTL2 lacked its transmembrane domain, the protein was localized exclusively in the nucleus. We hypothesize that under drought stress, BvNTL2 undergoes proteolytic cleavage, removing the transmembrane domain and enabling nuclear translocation and functional activation. However, the precise cleavage mechanism and the nature of the putative nuclear localization signal (NLS) remain unclear. Future studies will investigate the process of transmembrane domain cleavage under drought stress by employing proteases or protein phosphorylation.

Water status directly indicates drought tolerance in plants. Under drought stress, plants maintain metabolic balance by adjusting their stomatal apertures, altering osmotic potential, and enhancing antioxidant capacity. These responses are tightly regulated by complex signaling networks, with ABA as a central regulator [39,40]. Our results show that *BvNTL2*-overexpressing lines had higher relative water content, enhanced CAT, POD, and SOD activities, and lower electrolyte leakage. These traits suggest reduced membrane damage and improved drought resistance through water status maintenance and activation of the antioxidant defense system. In addition, the transgenic lines maintained higher chlorophyll content, stable Fv/Fm values, and favorable stomatal density and apertures, contributing to better water retention. These findings suggest limited damage to the photosynthetic apparatus, likely resulting from enhanced antioxidative capacity [41]. We speculate that this multifaceted adaptive mechanism results from cross-regulation of various signaling pathways. In contrast to previous studies on other NTLs, our results show that *BvNTL2* also enhances ABA signaling, as indicated by the markedly increased expression of *AtNCED3* and *AtAREB*, further supporting the key role of ABA in drought stress responses [42].

## 4. Materials and Methods

### 4.1. Identification and Prediction of the Physicochemical Properties of BvNTLs Family Members

Based on the 52 sugar beet NAC transcription factor genes identified in our previous research [27], DeepTMHMM (https://biolib.com/DTU/DeepTMHMM/, accessed on 7 February 2025) was used to predict that NAC transcription factor proteins containing at least one transmembrane domain are classified as NTLs [43]. The ExPASy ProtParam database (https://web.expasy.org/protparam/, accessed on 10 February 2025) was used to analyze the physicochemical properties of the proteins encoded by BvNTLs genes, including coding region length, amino acid count (aa), and theoretical isoelectric point (pI). The DeepLoc-2.1 online tool (https://services.healthtech.dtu.dk/services/DeepLoc-2.1/, accessed on 11 February 2025) was used to predict the subcellular localization of NTL gene family members [44]. The chromosomal locations of NTL genes were obtained from the genome annotation file and visualized using TBtools [45].

### 4.2. Gene Structure and Conserved Motif Analysis of BvNTLs

The MEME tool (https://meme-suite.org/meme/tools/meme, accessed on 11 February 2025) was used to analyze the conserved motifs of the NTL gene family proteins, with the maximum number of motifs set to 10.

### 4.3. Comparative Synteny and Phylogenetic Tree Analysis of BvNTLs

Phylogenetic and protein sequence clustering analyses of the NTL gene family members were conducted using MEGA 11 software (version 11.0.13). The iTOL online tool (https://itol.embl.de/, accessed on 11 February 2025) was used to edit and optimize the phylogenetic tree [46]. BLASTP alignment was used to identify orthologous gene pairs across multiple species. The MCScanX function in TBtools was used to identify syntenic blocks between species.

### 4.4. Cis-Regulatory Element Analysis of BvNTLs Promoters

The promoters of *BvNTLs* (2000 bp upstream of the translation start site) were obtained using TBtools. PlantCARE (https://bioinformatics.psb.ugent.be/webtools/plantcare/html/, accessed on 13 February 2025) was used to identify and locate cis-regulatory elements within each promoter.

### 4.5. Generation of Transgenic Arabidopsis Plants

The pCAMBIA1300-GFP-BvNTL2 construct was used as an overexpression vector and introduced into *Agrobacterium tumefaciens* strain GV3101. Transgenic *Arabidopsis thaliana* lines were generated using the floral dip method [47]. Hygromycin-resistant plants were screened, and homozygous lines were selected in the T3 generation for phenotypic analysis.

### 4.6. Plant Materials and Growth Conditions

The sugar beet variety HI0466, developed by Syngenta (Basel, Switzerland), was evaluated for drought resistance by the Sugar Beet Physiology Research Institute at Inner Mongolia Agricultural University (Hohhot, China). Seeds were sown in trays containing a 1:1 mixture of vermiculite and soil. Seedlings were cultivated in a controlled growth chamber under a 16/8 h light/dark cycle at 24/20 °C (day/night). Drought treatment began when the first pair of true leaves had fully expanded. Seedlings were first thoroughly watered, followed by a two-week period without irrigation.

### 4.7. Quantitative Real-Time PCR Analysis

Total RNA was extracted using the FastPure Plant Total RNA Isolation Kit (Polysaccharides & Polyphenolics-rich) (Vazyme, Nanjing, China). First-strand cDNA was synthesized from 2 μg of total RNA using the HiScript II 1st Strand cDNA Synthesis Kit (Vazyme, Nanjing China). Quantitative real-time PCR (qRT-PCR) was performed using the LightCycler^®^ 96 real-time PCR system (Bio-Rad, Hercules, CA, USA). All qRT-PCR analyses were performed with three biological replicates, using *AtACTIN-F*, *AtACTIN-R*, *BvACTIN-F*, and *BvACTIN-R* as internal reference genes. Primer sequences are provided in Supplementary Table S1.

### 4.8. Physiological Index Analysis of Transgenic Arabidopsis

Fresh leaves from WT and *BvNTL2*-overexpressing *Arabidopsis* plants were collected under both normal and drought conditions for physiological measurements. The measured parameters included relative water content [48], electrolyte leakage [49], and water-loss rate [50]. Chlorophyll was extracted with 80% acetone and quantified [51]. Malondialdehyde (MDA) was extracted using the Thiobarbituric acid (TBA) method [52]. Proline content was determined according to Bates et al. [53]. Antioxidant enzyme activities, including superoxide dismutase (SOD), peroxidase (POD), and catalase (CAT), were measured following Rao et al. [54].

### 4.9. Statistical Analysis

All experiments were independently repeated at least three times, and data are presented as the mean ± standard deviation (SD) of three biological replicates. Statistical analyses were conducted using SPSS software (version 22.0; SPSS Inc., Chicago, IL, USA). Analysis of variance (ANOVA) and Student’s *t*-tests were used to assess significance between samples, with *p*-values < 0.05 considered statistically significant.

## 5. Conclusions

In this study, five *BvNTLs* gene family members were identified, each located on a different chromosome. The encoded proteins range from 383 to 608 amino acids in length, with isoelectric points (pIs) between 4.57 and 8.70. All proteins are predicted to be hydrophilic, and most are localized to the nucleus based on subcellular localization analysis. All *BvNTLs* proteins contain more than seven conserved motifs, including NAM and transmembrane domains. Forty-two distinct cis-acting elements were identified in the promoter regions, primarily related to plant hormone and abiotic stress responses. Synteny analysis revealed conserved genomic regions across species, and phylogenetic analysis indicated that *BvNTLs* are closely related to homologs in *Arabidopsis thaliana* and *Solanum lycopersicum*. Functional analysis in transgenic *Arabidopsis* showed that *BvNTL2* enhances drought tolerance by upregulating stress-responsive genes, resulting in increased antioxidant enzyme activities (SOD, POD, and CAT), higher chlorophyll content, and elevated proline (Pro) levels. Moreover, *BvNTL2* expression reduced hydrogen peroxide (H_2_O_2_), malondialdehyde (MDA), and superoxide anion (O_2_^−^) levels, while decreasing stomatal conductance and electrolyte leakage. These findings suggest that *BvNTL2* enhances drought tolerance by activating antioxidant defenses and improving water retention. This study presents the first systematic characterization of the NTL gene family in sugar beet and functionally validates *BvNTL2* in the context of drought response, offering new insights into membrane-bound NAC transcription factors in a root crop species.

## Data Availability

Data are contained within the article and Supplementary Materials.

## Supplementary Materials

The following supporting information can be downloaded at https://www.mdpi.com/article/10.3390/plants14101528/s1, Table S1: Primers used in the experiment; Table S2: Physicochemical properties; Text file S1: *BvNTLs* family gene sequences; Text file S2: Protein sequences required for constructing the phylogenetic tree.
