# Supplementary material for: Identification of the NTL Gene Family in Beta vulgaris L. and Functional Role of BvNTL2 in Drought Resistance
Source: Plants (Basel). 2025 May 20;14(10):1528. doi: 10.3390/plants14101528 (PMC12114746; doi:10.3390/plants14101528)
Supplement: Supplementary file 1 [file plants-14-01528-s001.zip › Physicochemical Properties.pdf]

Name	Gene ID	Number of Amino Acids	Molecular Weight	Theoretical pI	Grand Average of Hydropathicity	Subcellular Localization
BvNTL1	BVRB_1g022030	388	43840.19	8.70	-0.455	Nucleus
BvNTL2	BVRB_2g032870	393	44094.95	8.54	-0.540	Nucleus
BvNTL3	BVRB_3g050780	608	67932.70	5.77	-0.525	Nucleus
BvNTL4	BVRB_4g093720	540	59948.87	4.57	-0.404	Endoplasmic reticulum
BvNTL5	BVRB_8g199060	527	58918.33	4.85	-0.509	Nucleus
