# Supplementary material for: Identification of the NTL Gene Family in Beta vulgaris L. and Functional Role of BvNTL2 in Drought Resistance
Source: Plants (Basel). 2025 May 20;14(10):1528. doi: 10.3390/plants14101528 (PMC12114746; doi:10.3390/plants14101528)
Supplement: Supplementary file 1 [file plants-14-01528-s001.zip › Primers Used in the Experiment.pdf]

AtACTIN-F	CTGGATTCTGGTGATGGTGTGTCT
AtACTIN-R	GAACCACCGATCCAGACACTGTAC
AtNCED3-F	CAAGTTCAGATCACTCCCAA
AtNCED3-R	GCTTAAAGCGAAGAGTTCAC
AtAAO3-F	GGAGTCAGCGAGGTGGAAGT
AtAAO3-R	TGCTCCTTCGGTCTGTCCTAA
AtABA1-F	GGCATTTGGTCTAAGGTGAGAA
AtABA1-R	CAGACTCGATCCGCTGGTA
AtABA2-F	TTCTCTTCCTAGTCAAAGGCTTT
AtABA2-R	GCAGACTTTGGCACCGTGCT
AtABA3-F	CAAAAGGAAGAGTCAAGAGGAA
AtABA3-R	TTTCTTTCATCAACTTCACCAGAT
AtAREB-F	GGTAACATTGTCTCAGTGGTGG
AtAREB-R	AACGACCTTAATCTTCAGCTGC
ATDR4-F	CGTCCTGGTCAAACGTACCA
ATDR4-R	TCGGAGCCTCAAACTCGATG
AtMDAR-F	GTACACCGGTTTTTCACCGC
AtMDAR-R	CCGACACCAGCGACAACTAT
AtSOD-F	ATGAGAAGTTCTATGAAGAG
AtSOD-R	GTCTTTATGTAATCTGGT
AtCAT-F	GCAACTACCCCGAGTGGAAA
AtCAT-R	TGTTCAGAACCAAGCGACCA
AtP5CS1-F	GCGCATAGTTTCTGATGCAA
AtP5CS1-R	TGCAACTTCGTGATCCTCTG
AtPOD-F	GAGGAGAAGGCCAGGAAAGG
AtPOD-R	CCAGTGAAGACGAGGTGGAA
BvACTIN-F	TGCTTGACTCTGGTGATGGT
BvACTIN-R	AGCAAGATCCAAACGGAGAATG
BvNTL2-F	ATGTTCCCAGGGTTTAG
BvNTL2-R	ATCCCAAGGTTCGTATT
